# P4s Are Either Unhelpful or Unnecessary. Proposing a Better AI‐Powered Solution to Predict Patients' Preferences

**DOI:** 10.1111/bioe.70114

**Published:** 2026-05-07

**Authors:** Beatrice Marchegiani

**Affiliations:** ^1^ University of Oxford Oxford USA

**Keywords:** advance directives, AI, personalised medicine, personalised patient preference predictors, surrogate decision making

## Abstract

The *Personalized Patient Preference Predictor* (P4) has been proposed as an AI tool to aid surrogate decision‐making when incapacitated patients lack advance directives. Unlike population‐level *Patient Preference Predictors* (PPPs), which infer preferences from demographic correlations, P4s fine‐tune large language models (LLMs) on a patient's digital footprint to simulate their likely treatment preferences. The goal is to preserve autonomy by grounding predictions in individualized data rather than broad statistical trends. This paper argues that P4s face a fundamental dilemma: they are either unnecessary or unhelpful. When relevant, individualized evidence of preferences exists in a patient's digital footprint, the P4 is unnecessary and suboptimal, since the original data can be examined directly, with richer context than a generative model can preserve. When no such evidence exists, the P4 is unhelpful or misleading, producing plausible‐sounding outputs that in the best case rely on population‐level correlations rather than the patient's own values. To address these limitations, I propose a better AI‐powered alternative: the *Patient Preference Retriever* (PPR). Rather than generating new text, the PPR uses vector search techniques to retrieve relevant statements from a patient's digital footprint, presenting them verbatim alongside metadata such as date, context, and source. This approach offers greater transparency, respects autonomy more reliably, and supports surrogate decision‐makers in weighing authentic evidence. I conclude that while advance directives remain the gold standard, retrieval‐based approaches like the PPR provide a more reliable and ethically defensible use of AI in surrogate decision‐making than generative approaches like P4s.

## 1 Introduction

In clinical practice, it is not uncommon to encounter patients who are unable to communicate their preferences and who have not left behind any advance directives. In such cases, healthcare professionals and families are confronted with a difficult question: What would this patient have wanted? Determining the right course of treatment becomes ethically and emotionally fraught, especially when time is limited and uncertainty is high.

A potential solution suggested in the literature is to design an algorithm capable of predicting patients' preferences, a Patient Preference Predictor (PPP), which works by making statistical inferences based on data from other patients with similar characteristics by using population‐level demographic correlations [1]. Several authors have argued that PPPs face a range of serious problems [2, 3]. A key limitation of PPPs is that they threaten the patient's autonomy: decisions about a medical intervention should be guided by factors that reflect the individual patient's own values, rather than relying on broad population‐level correlations, such as those based on demographics like race, age, or socioeconomic status.

In response to this worry several alternative predictors have been proposed^1^: the most recent is the Personalised Patient Preference Predictor (P4) provided by Earp et al [5]. P4s are large language models (LLMs), fine‐tuned on a patient's digital footprint (such as their social media use, blog posts, emails, purchase and browsing history, and so on), to approximate what that patient would have said in cases where they are no longer able to express their preferences. The idea is that, unlike the PPP, the P4 bases its decision‐making on individualised evidence, not mere statistical correlation, thereby protecting patients' autonomy. Here, individualized evidence should not be understood merely as information about the patient; rather, it must be personalized in the stronger sense of directly capturing the patient's own expressed beliefs, values, and commitments. Several scholars have already raised objections to the P4 approach. Blumenthal‐Barby et al. [6] question what kinds of data should be used as a basis for prediction, noting that some sources may distort rather than clarify patient preferences, and express doubt that even the best‐quality data could yield reliable predictions or meaningfully ease the burden on the patient's surrogate decision makers. Rzepiński et al. [7] highlight that many important aspects of patient preference are communicated through nonverbal means (such as voice tone, gestures or expressions) that P4 systems are unable to capture in their training data. Sharadin [8] draws attention to the instability of P4 outputs, showing that different prompts can produce different results, further undermining their reliability. Mertes [9] argues that the P4 is misleading in claiming to make reliable individual‐level predictions, since AI can only generate probabilistic group‐level inferences, which risk misrepresenting a patient's actual preferences. Improving the P4 predictive power would require extensive data‐gathering efforts that are more burdensome than simply encouraging advance directives. Similar concerns have also been raised by Battisti [10], who argues that instead of attempting to predict the preferences of already incapacitated patients, AI should be used to support patients in creating advance directives. Lastly, Annoni [11] has questioned whether P4s actually protect user autonomy.^2^

In this paper, I argue that P4s are either unnecessary or unhelpful at achieving their goals of predicting patients' preferences based on individualised data. There are exactly two scenarios: either the patient's digital footprint contains data which indicates their preferences or it does not. If the data is present then P4 is unnecessary (and suboptimal). If the data is not present then the P4 is unhelpful (and possibly misleading). When a P4 can meet its goal, this is only possible because there is data expressing the patient's preference in the patient's digital footprint. In such cases, we would be better off directly retrieving and interpreting this first‐hand information, rather than relying on a predictive model that adds potential noise. When a P4 cannot meet its goal (i.e., when there is little or no individualized data relevant to the specific medical choice), the tool becomes unhelpful or even misleading as it will still generate an output, thus giving a false impression of reliability.

In light of these limitations, I propose a better AI‐driven solution: the Patient Preference Retriever (PPR). This approach uses a combination of LLMs and vector search methods to navigate a patient's digital footprint and extract pieces of information that are directly relevant to the clinical decision at hand. Its output is a curated set of data points from the patient's own digital footprint, categorized as either supporting or opposing a given medical decision. The final decision remains with the patient's next of kin, in consultation with the clinicians, who together determine how to interpret and weigh this evidence in light of the clinical context.

This paper is structured as follows. In Section 1, I examine the technical foundations of P4s, focusing on how fine‐tuned LLMs operate. In Section 2, I develop the central dilemma: when relevant, individualized preference data exists in the patient's digital footprint, P4s are unnecessary and suboptimal; when such data is absent, they are unhelpful and potentially misleading. I also discuss how the manner of presentation (e.g., mimicking the patient's voice) reinforces this dilemma. In Section 3, I illustrate the dilemma in practice through a hypothetical case study. In Section 4, I introduce the Patient Preference Retriever (PPR), a retrieval‐based alternative that uses vector search to surface verbatim statements from a patient's digital footprint. I describe how the system could be implemented and compare it to P4s along four key dimensions: transparency, respect for autonomy, privacy, and clarity of decision‐making. Finally, I conclude that while advance directives remain the preferred option when it comes to respecting patient autonomy, retrieval‐based approaches like the PPR offer a better way of integrating AI into surrogate medical decision‐making than generative approaches like the P4.

## 2 Personalisation Through Fine Tuning: What P4s Want to Predict V What They Actually Predict

Given that P4s are proposed to be implemented through fine‐tuned LLMs, it is important to first understand how LLMs work, and specifically, how personalization through fine‐tuning operates. At their core, LLMs are statistical models that predict the next token in a sequence. A token can be a single word, part of a word or a group of words. Given a sequence of tokens, the LLM estimates the most likely next token, based on the statistical distribution it learned from a massive corpus of training data. The model, trained on general text from the internet, books, articles, and other sources, is designed to reflect broad linguistic patterns across many domains. Fine‐tuning is a process in which this base model is further trained on a smaller, more specific data set to adjust its behavior. In practical terms, fine‐tuning changes the statistical distribution of the model's outputs. It shifts the likelihood of certain tokens occurring, based on the patterns found in the fine‐tuning data. When the data a model is fine‐tuned on is someone's digital footprint, this allows the model to mimic an individual's communication style and semantic patterns. Imagine we fine‐tune a base LLM on a person's emails, blog posts, and social media content and so forth. After fine‐tuning, the model will adopt their tone, vocabulary, recurring phrases and common views (given that they are present in the data set).

Applying this in the context of P4s, what the model does is, given a sequence of input tokens, predict the most likely next token, assuming that the sequence is drawn from the same statistical distribution as the patient's digital footprint. The idea behind P4s is that by fine‐tuning on a patient's digital footprint, the model can generate outputs that reflect what that specific patient (P) would likely choose in a given medical decision (X). The goal of a P4 can be divided into two criteria:

- [Accuracy]: the output should be as close as possible to what P would actually have chosen, and
- [Justification]: the output should be grounded in individualized evidence drawn from P's own digital footprint, rather than from population level statistical correlation. This is what differentiates the P4 from the PPP and according to Earp et al. [5] ensures that the P4 respects the patient's former autonomy when direct autonomous decision by P is no longer possible.

P4 can satisfy [Accuracy] only if we assume that generating text with a similar token distribution to P's prior communication will yield an output that accurately reflects her preferences in X. But these are different tasks. Sharadin [8] argues that the task of generating text using AI models to predict a patient's preferences is fundamentally different from the task of predicting their medical choices. It is generally unjustified to assume that similarity in token distribution (a computational task in text generation) can effectively translate into accurately understanding and predicting a patient's medical choices. When, then, can the outputs of next‐token prediction genuinely track the kind of preference information P4s are meant to provide? There are two possible cases:

- Case 1: Relevant individualized data is present in P's digital footprint. If P's digital footprint contains enough clues and relevant information about the patient's medical preferences then next token prediction *should* be able to surface this information in the P4 output. Here “relevant” means data that bears directly on the decision at hand (e.g., prior statements about treatment preferences, reflections on risk tolerance, or discussions of religious or cultural values that shape medical choices). In such cases, the model's output is not only more likely to be accurate (satisfying [Accuracy]) but also grounded in P's own individualized data (satisfying [Justification]).
- Case 2: Even without relevant individualized data, the P4 might sometimes predict P's choice correctly. This could happen either by complete chance or if P happens to align with correlations the base model has learned during training. For instance, if the training data for the base model reveals a statistical correlation between people who order Chinese food, watch horror movies, and those who would refuse life support in the event of severe brain damage, the model could infer from a patient's unrelated digital footprint (evidence that they frequently order Chinese takeout and stream horror films) that this patient too would decline life support. If P happens to fit that pattern, the prediction may align with her actual choice. However, such a result is empirically questionable at best. Even if the base model had learned these correlations, many of them may not form a reliable basis for statistical inference. Research shows that even state‐of‐the‐art LLMs (the models that would serve as the base model for P4s) are trained on data sets that are biased and not culturally representative [13, 14, 15, 16]. This means we cannot assume that statistical correlations in the base model are trustworthy, given the bias present in the training data. Even putting these concerns aside, if the result from the P4 were accurate, such success would rely on generic population‐level correlations rather than P's own values. This issue is particularly pressing because it collapses the P4 back into the same strategy as the PPP, which relies on socio‐demographic data to predict what the patient might choose. While the P4 might appear to avoid some of the autonomy concerns of the PPP, when relevant data is absent, it still fails to base decisions on the individual patient's own values. Instead, it relies on the assumption that people with certain shared characteristics make similar decisions. In this way, the P4's reliance on correlations learned from large data sets brings us back to the same issues of autonomy that are present in the PPP, which the P4 was purported to avoid. The absence of relevant individualised data is ethically problematic, since it fails the [Justification] criteria.^3^

Thus, the P4 can only meet both of its desiderata [Accuracy] and [Justification] if relevant individualized information is present in the patient's data. This is the stronger, autonomy‐preserving case, and it is the one I analyze in the remainder of the paper.

Before proceeding I want to address a recent claim by Earp et al. [17]^4^ arguing that there is growing empirical evidence that LLMs can accurately infer personal preferences and take this as supporting the claim that P4s can meet the [Accuracy] criteria. They cite a study by Park et al. [18] where LLM‐based agents correctly inferred preferences 84% of the time when given access to interview transcripts. However, the technical implementation of the agents in that study differs radically from the P4 proposal. In Park et al., the user's information is not used for fine‐tuning; instead, the interview transcript is injected directly into the model's prompt. This distinction is important for two reasons. First, this ‘prompting’ method does not scale to the P4 use case. While a single interview can fit into a system prompt, years of a user's digital footprint would likely exceed the token limit of current models. Second, because the model in the study only achieved 84% accuracy with the data directly in front of it, it is unlikely that a fine‐tuned P4 model would perform better. In fact, relying on next‐toxen prediction based on fine‐tuning rather than direct access to transcripts would almost certainly result in lower accuracy. Consequently, it is difficult to see this study as evidence for the accuracy of P4 models.

## 3 The Unnecessary and Suboptimal/Unhelpful and Misleading Dilemma

In the previous section, I clarified that what a P4 actually predicts (most likely pattern given token distribution in a patient's digital footprint) and what it needs to predict (patient's medical preference) are fundamentally different. Yet despite this, the hope behind P4s is that, by fine‐tuning on data of sufficient quality and relevance, simply replicating linguistic patterns in the fine‐tuning data will produce outputs that align with a patient's preferences. Note however that, if P4 can predict patient preference only when the patient's digital footprint contains relevant information then P4s are either unnecessary and suboptimal or unhelpful and potentially misleading. This is because when relevant data is present, it's better and more accurate to examine it directly, and when relevant data is absent, the model generates plausible‐sounding guesses that risk misrepresenting the patient's preferences. When I state that direct examination is better I am not suggesting that manual, direct examination of patient's data is a feasible alternative. I propose an alternative AI system capable of efficiently retrieving the relevant data for direct analysis, as opposed to the P4, which generates new responses based on the data. This solution will be explored further in section 5, after I have fully addressed the dilemma of P4s being either unnecessary or unhelpful.

### 3.1 Unnecessary and Suboptimal

Suppose the patient's digital footprint contains sufficient relevant individualised information to accurately predict the patient's medical decision. Note that in this context, relevant individualised data cannot be understood in the weak sense of merely being data about an individual (for instance, knowing that this particular patient belongs to a certain demographic group, which might improve statistical inference but does not capture their own values and thus doesn't protect their autonomy). Instead, it must be individualised in the stronger sense of directly reflecting the patient's expressed beliefs or commitments. For example, this might include explicit discussions with family members about what they would want in certain circumstances, a formal record of being registered as an organ donor, or consistent medical records that reflect a clear stance on similar issues. In that case, the next‐token predictor can incorporate this into its output because the statistical patterns in the training data align with the preference we're trying to predict. When such data is present, what the P4 predicts and what it should predict converge. So in this situation, the P4 is able to produce a useful output. However, note that the usefulness of the P4 is due entirely to the quality of the underlying data. So even if the output is useful, the P4 itself is unnecessary. We could determine what the patient would have wanted simply by directly examining the relevant available evidence. There is no need for a model to represent this information. On closer examination P4s are not just unnecessary, they are also suboptimal due to the loss of valuable metadata through the fine‐tuning process. When the patient's data (say a message in an online chat) is used to fine‐tune a model, important contextual details (such as who the patient was speaking to, when the conversations happen, or whether the comment was serious or joking) are stripped away. To the model, a sequence of tokens is just that: a sequence of tokens. It makes no difference whether a statement was said jokingly, in a moment of frustration, in private, or many years ago, all sequences of tokens are weighted the same, and the model simply picks up statistical correlations between tokens. In the context of a serious medical decision, making a choice based on something the patient once said, without considering the broader context in which it was said, is suboptimal. The P4 acts as an unnecessary and unreliable intermediary, like playing a game of broken telephone. It would be far better to present the original data directly, so that those making the decision (doctors and next of kin) can assess its relevance, consider the circumstances in which it was expressed, and weigh it accordingly in the context of the current medical decision. In the original proposal for the P4, Earp et al. suggest giving greater weight to significant data during fine‐tuning by training on it for multiple epochs [5]. But this raises a basic question: if we already know which data is significant, then we can simply examine that data directly, without needing to filter it through a language model. As a heuristic, Earp et al. [5] suggest that we should prioritise data based on its source, for example by prioritising medical records and personal communications over social media interactions. But there is no guarantee that such data is actually the most relevant or reliable for the specific decision at hand.

### 3.2 Unhelpful and Misleading

In all other cases, when the patient's digital footprint lacks relevant individualized data, we have no reason to expect that the P4's output will reflect the patient's true preferences. What the model will reliably reproduce is the stylistic and linguistic patterns found in its training data. So the output might resemble the communication style of the patient. However, stylistic resemblance does not mean the content reflects the patient's actual medical preferences. In the absence of relevant patient data in the fine‐tuning step, the model fills in gaps using patterns from the base model, which are derived from broad population‐level trends. So even if the P4 happens to guess what the patient would have decided, it does so by relying on population‐level statistical patterns learned from the base model. In this way, the P4 is effectively reverting to the same strategy used by PPP (recall the discussion in Section 1). If the goal is to determine the patient's preferences based on individualized evidence, then in the absence of useful individualized data the P4 is ultimately unhelpful. Worse still, the P4 is also potentially misleading. In the case of a PPP, it is clear that any output is based on broad statistical trends. This transparency helps manage expectations. By contrast, a P4 presents itself as fine‐tuned on the patient's own digital footprint.^5^ From the outside, there is often no way to tell whether a given output reflects genuinely relevant individualized data or whether it is merely a plausible‐sounding guess filled in by the base model. This is particularly concerning in high‐stakes medical situations, where those making decisions may lack technical understanding of the model limitations and may be in an emotionally compromised state. As a result, they may wrongly assume that *all* P4 outputs are grounded in individualized evidence, when in fact, they are not.

Once a model is fine‐tuned, the fine‐tuning data becomes diffused within the model's parameters. Just by looking at a P4's output, it is practically impossible to tell whether it reflects genuine individualized data. Researchers in machine interpretability are developing techniques to trace outputs back to training data. However, current methods don't apply well to the P4 use case. Gradient‐based and influence function approaches [19] require high computational resources, especially when run over large token sets. Other interpretability methods do not perform well if the model's output is generated using paraphrased information (rather than verbatim quoting from the training data) [20]. As a result, there is currently no reliable way to trace a P4 output back to a specific training example in a patient's digital footprint. This means we can't tell whether a given output is grounded in relevant patient data or synthesized from general patterns in the base model. This uncertainty worsens the dilemma: it makes P4 outputs even more suboptimal when they are based on relevant information, since we cannot verify it, and even more misleading when they are not, as we might falsely believe they are.

### 3.3 The Manner of Presentation Further Reinforce the Suboptimal/Misleading Dimension

While the original proposal for the P4 does not specify exactly how the output will be presented, the authors' emphasis on fine‐tuning a LLM using personal text generated by the patient suggests that one plausible implementation would be to have the output mimicking the style and voice of the patient, effectively creating a kind of digital twin. I want to briefly discuss the negative impact of this manner of presentation. Suppose the P4 adopts the patient's tone, vocabulary, and even uses the first person. Depending on the contents of the patient's digital footprint, it might generate statements like, “I don't want to be kept alive as a vegetable, please just let me go,” or “Please keep me alive, I'm scared of dying.” This can be deeply unsettling, especially when the people reading these outputs are not only clinicians, but the patient's family and loved ones who recognise the patient's characteristic conversational style in the P4 output. Beside being a source of emotional distress, presenting the P4 output in the style and voice of the patient reinforces the suboptimal/misleading dimension of the dilemma. If relevant evidence *is* present, presenting it in the patient's voice adds an unnecessary layer of emotional intensity, which can obscure the evidentiary value of the content. If no such evidence exists, then simulating the patient's style is actively misleading. It gives the false impression that the model's output reflects the patient's wishes, simply because it *sounds* like them. Note that there is no technical requirement that P4s present outputs in the patient's voice. An intermediate model could rephrase the output in a neutral tone. This wouldn't solve the fundamental unnecessary/unhelpful dilemma, but it would mitigate the problem discussed in this paragraph. All we are interested in is identifying if there is relevant information in the patient's digital footprint, there is no reason why decision‐relevant evidence should be presented in the style and voice of the patient. Moving on I will set the issue of manner of presentation aside.

## 4 The Unhelpful/Unnecessary Dilemma in Practice

To further argue for my claim that P4s are either unnecessary and suboptimal or unhelpful and potentially misleading let's examine a practical scenario.

[Alice] Suppose Alice was in a car crash. She is now unconscious and brain damaged. If she was provided with life support she might survive, but given the extent of her injuries she would be completely paralysed for the rest of her life. We want to know what her preference would be regarding being kept on life support. She has not left any advance directive.

There are three possibilities to consider.

### 4.1 Clear Decision‐Relevant Information Is Present in the User's Data

Consider the case where there is clear evidence in her digital footprint that Alice did not want to be kept on life support. Suppose that about a month before her accident, Alice chatted via a messaging app with one of her closest friends about a recent “right to die” case in the news. At one point, Alice stated clearly: “I can't think of anything worse than being paralysed, with severe brain damage and hooked to a machine.” Given this data, a fine‐tuned P4, trained on her digital footprint, outputs: “Alice wouldn't want to be kept on life support.” The P4 output reflects a clear, pre‐existing preference stated directly by Alice. However, the model is not doing anything that could not be done by simply retrieving and quoting that original message. In this case, the P4 is accurate, but also unnecessary. In fact, relying on the P4 is suboptimal. By examining the original conversation, one would see not just the statement but its rich context: the fact it was recent, that it was shared with a trusted friend, and that it was part of a long, thoughtful discussion. All of this context would be stripped away and obscured by the P4.

### 4.2 No Decision‐Relevant Information Is Present in the User's Data

Suppose Alice's digital footprint contains no mention of medical preferences, end‐of‐life care, religious beliefs, or related values. Despite this, the P4 produces the output: “Alice wants to stay on life support.” This statement is based on patterns in the base model's training rather than any individualized evidence from Alice's past communications. There is no personalized foundation for the claim, yet it is presented with the same authority as an personalised evidence‐based answer, creating a false sense of reliability. In this case, the P4 is not only unhelpful but misleading.

### 4.3 Conflicting Decision‐Relevant Information Is Present in the User Data

Finally, consider the case where Alice's digital footprint contains mixed evidence. This is the most realistic scenario because humans are not static. They evolve, change beliefs, and sometimes feel uncertain. They may even hold conflicting or contradictory views. Suppose that for most of her life, Alice belonged to a church that teaches withdrawing life support for paralyzed individuals is a sin. As such this position appears in a large number of data points in Alice's digital footprint (both in private conversations with her family and in public social media posts). However, in more recent private conversations with a close friend, Alice expressed doubts about her faith and began rethinking her views on withdrawing life support. Despite these evolving thoughts, she continued to express her earlier views to her family out of fear of judgment. How would a P4 handle such conflicting beliefs? The model is more likely to reproduce views that occur more frequently in the data set, even if those views are outdated or no longer representative. That's a statistical property of a LLM trained via next‐token prediction. Because the model looks for statistical patterns in tokens, the far greater number of data points supporting her earlier opposition to withdrawing life support would dominate. Therefore, the P4 would output: “Yes, I would want to stay on life support.” Fine‐tuning treats all data as equally relevant. It does not account for timing, context, or the evolution of beliefs. As a result, the P4 risks suppressing Alice's most reflective or recent statements, producing a dangerously misleading result. The original P4 proposal suggests giving some data sources more weight during training by running extra epochs on it [5]. The P4 proposal's heuristic of weighting “important” sources or recent datapoints more heavily doesn't help when conflicting statements come from equally weighted sources and were made around the same time. In Alice's case, both statements occur in personal conversations with close family or friends, meaning they would be weighted equally. This makes it likely that the P4 would still ignore the doubts Alice expressed to her friend and instead reflect the sheer volume of anti‐withdrawal of treatment statements in her digital footprint. When a data set contains conflicting evidence, the model will tend to favor the view that appears most often, even if it no longer reflects the patient's actual beliefs. In such cases, the P4 risks producing a misleading result.

## 5 A Better Alternative—The Patient Preference Retriever

So far, I've argued that the P4 is either unhelpful for determining patient preferences or unnecessary, offering no clear benefit over reviewing the patient's digital footprint directly. A possible defense of the P4 is that while direct analysis of raw data may be ideal, the sheer volume of the patient's digital footprint makes manual review infeasible. If that's the case, then the P4 might be the best way to navigate and extract relevant information efficiently. I disagree. In this section, I propose an alternative: the Patient Preference Retriever (PPR). Unlike the P4, which generates outputs offering no transparency about where its claims come from, the PPR is designed to surface actual statements the patient made, verbatim, and directly link each one to its original utterance. Instead of guessing what the patient *might* say, the PPR presents documented evidence of what the patient *did* say. Here is how it might work in the case of [Alice]. A clinician asks the PPR: “Would the patient want to be kept alive with severe brain damage and extremely low chance of recovery?” The system searches Alice's digital footprint and returns relevant excerpts either supporting or opposing the medical decision, each accompanied by metadata. Each statement is presented verbatim, along with its date, context, and a link to the original source [Figure 1]. The clinician together with Alice's next of kin can then evaluate the relevance and reliability of the evidence. The PPR does not reinterpret Alice's voice, instead it retrieves and presents what she actually said. This transparency and traceability sharply distinguish the PPR from the P4. In the following Section 5.1 will compare the P4 to the PPR, showing that the PPR has both technical and ethical advantages.

**Table:** 

Query: *Would the patient want to be kept alive with severe brain damage and extremely low chance of recovery??*
Search Result: *Searched Alice's digital footprint for evidence relevant to this question. Here are the statements found:*
Statements suggesting she may not have wanted life‐sustaining treatment: “I've always said I wouldn't want to live hooked up to machines. That's not living.” — Source: Email to sister, July 2021 [LINK] “My biggest fear is ending up in a coma and not being able to communicate. Please don't let that happen to me.” — Source: Group chat with friends, March 2019 [LINK] Signed up for organ donation. — Source: Email confirmation from National Donor Registry, August 2020 [LINK] Statements suggesting she may have wanted continued treatment: “I don't want to leave my children alone.” — Source: Message to partner during illness scare, November 2020 [LINK] Commenting on a news story about a woman who made a miraculous recovery from being paralysed: “Can you imagine it being me? The worst thing in the world would be pulling the plug if there's still hope.” — Source: Facebook comment, January 2021 [LINK] “My faith is a big part of who I am.” — Source: Personal introduction email to new colleague, September 2018 [LINK]

A technical high‐level overview of a potential PPR implementation using a data searching technique known as vector search is provided in Appendix 1.

### 5.1 Compare PR With P4

Having proposed a feasible alternative to the P4, I now turn to a direct comparison to highlight the differences and show that, given the goal of predicting what an incapacitated patient would choose in a medical decision based on individualised information, the PPR should be preferred. Table 1 presents a side‐by‐side comparison of the P4 and the PPR. In almost every category, the PPR performs as well as or better than the P4. Four dimensions stand out as particularly important for closer examination: transparency, respect for user autonomy, privacy, and clarity of decision.

**Table 1:** Side‐by‐side comparison of P4 (Generative) vs. PPR (Retrieval) approaches in patient decision‐making support, including technical behavior and ethical considerations.

Aspect	P4	PPR
High‐level description of how they work	Generative approach. Fine‐tuned LLM. Model parameters are updated using the patient's data. The model learns statistical token patterns and generates an output. The hope is that with enough relevant data the P4 can mimic the patient's decision‐making patterns.	Information retrieval approach (e.g., Vector Search). The system searches for semantically relevant items to the query and returns them directly. The emphasis is on surfacing data authored by the patient that supports or opposes the decision at hand.
Behavior with no relevant data	× Unhelpful and potentially misleading.	× May return no results or unrelated data points, but avoids generating confident false outputs.
Behavior with decisive relevant data	✓ Unnecessary and suboptimal.	× Returns the supporting evidence verbatim, linking to the data.
Behavior with mixed relevant data	✓ Amplify the more frequent opinions present in the training data, which may misrepresent the patient's actual preference.	× Presents both pro and con data transparently, allowing decision‐makers to weigh evidence themselves.
Manner of presentation of answer	✓ Outputs a single statement phrased as the patient's preference. Depending on implementation risks speaking in the patient's voice.	× Impersonal; focuses on evidentiary content, returning one or more source excerpts.
Transparency	✓ Low: underlying source data cannot be traced once fine‐tuned.	× High: each output is directly linked to original evidence, with date, context, and source.
Patient's autonomy	⚠️ Partially supports autonomy by drawing on patient data, but its opacity makes it unclear whether outputs reflect individualized evidence or generic statistical patterns, risking misrepresentation of preferences.	× Better support of autonomy by directly retrieving the patient's own recorded statements, preserving the original context and ensuring decisions are grounded in authentic prior expressions.
Privacy (both patient and third‐party)	✓ Sensitive information may be inferred but is not explicitly exposed; some indirect risk to third parties.	✓✓ More vulnerable. Data is surfaced verbatim, potentially exposing sensitive patient details and third‐party information contained in conversations.
Clarity of Decision	✓ Provides a single, definitive output, offering the appearance of certainty.	⚠️ Provides lists of supporting/opposing statements. Greater scope for interpretation by clinicians/family when there is mixed or inconclusive data.

#### 5.1.1 Transparency

When it comes to transparency, PPRs hold a clear advantage over P4s. Because PPRs link their outputs directly to the patient's original data, they enable a level of verifiability that P4 cannot offer. Recall that for the P4 it is not technically possible to trace an output to a specific point in the patient data set. While for the PPR decision‐makers can see exactly which statements or behaviours informed the result. This reduces the risk of misinterpreting or misrepresenting the patient's preferences. By contrast, P4s operate as opaque predictive systems, leaving it uncertain whether their outputs are grounded in individualized evidence. While PPRs are not flawless (relevant information might occasionally be missed through vector search) this limitation can be flagged to users, ensuring that the possibility of incompleteness is transparent. The added transparency of a retrieval‐based approach like the PPR over a generative approach like the P4 is especially relevant in medical applications, where decisions should be made with as much context as possible, and where avoiding false beliefs about a patient's preferences should be the primary goal.

#### 5.1.2 Respect for Patient's Autonomy

Whether P4 can respect the (former) autonomy of the patient is a matter of debate. In this paragraph I provide an overview of the debate, I do not take a side. Instead my aim is to show that if one holds that a P4 can respect patient autonomy, then a PPR would do so even better. Annoni [11] argued that even if a P4 succeeds in accurately predicting a patient's preferences, this does not suffice for autonomy. Autonomy, he insists, is not merely about arriving at the “right” treatment outcome but also about the process of participating in decision‐making. Earp et al. accept this point but push back against Annoni's criticism. They argue that P4s do more than generate statistical predictions: they “draw directly from the patient's own recorded expressions and behaviours.” and therefore enable a “more accurate connection to the patient's authentic prior values” [12]. In their view, the fact that P4 utilises individualised evidence from the patient (see my earlier discussion of what individualised means), enables P4s to preserve elements of the patient's unique identity and agency even when the patient can no longer decide for themselves. For Earp et al. it is precisely this ability to draw on individualized data that allows P4s to respect former autonomy. If one is to accept their defence then I argue that PPR are even better suited at respecting the patient's (former) autonomy. As I have shown, the opacity of P4 models makes it difficult to determine to what extent any given output was genuinely based on individualized evidence rather than broader statistical patterns embedded in the base model. If respecting autonomy requires grounding clinical decisions in the patient's own prior expressions of values and preferences, then PPRs perform this task more reliably. Unlike P4s, PPRs retrieve information directly from a patient's digital footprint, presenting it in a way that remains faithful to how the patient originally expressed it. Thus, while P4s may respect autonomy to some degree (by basing its output on the patient's data), their opacity makes them less trustworthy in this regard than PPRs.

Annoni [21] finds Earp's et al. [12]^6^ reply unconvincing. He argues that to respect the patient's autonomy is not enough to correctly identify the patient's preferences, rather what matters is that those “preferences were directly expressed by the patient through some specific act of self‐determination”. Note that Annoni might extend his criticism to PPRs as well. According to him, preferences expressed in the patient's digital footprint are not necessarily acts of will, like advance directives, but rather attitudes, inclinations, or dispositions that do not automatically translate into a binding medical decision. Thus, he might argue that respecting preferences expressed in the digital footprint (even when they are reported verbatim, as the PPR does) does not equate to respecting the patient's autonomy in the fullest sense.^7^ In response, I argue that even if we assume Annoni is correct and the PPR cannot fully preserve autonomy in every case, it still represents an improvement over the P4. There may be specific instances where a patient has directly expressed their preferences in a way that meets Annoni's requirements for self‐determination. In those cases, the PPR can surface that information. For example, imagine a patient that had a lengthy exchange with a close friend over text messages and reached the conclusion that they want to be an organ donor. This to me counts as a genuine act of self determination, and by bringing this exchange to light a PPR would ensure that the patient autonomy is preserved.

While I grant that advance directives are the best way to respect autonomy, in the absence of them, we must find a method that does the best it can. If Annoni's argument is correct and P4 models fail to preserve autonomy, then the PPR is at worst equal to the P4. However, the PPR may be superior in some cases because it can surface genuine acts of self‐determination if they exist within the data. Conversely, if one finds Earp et al.'s arguments convincing, the PPR appears even better at preserving patient autonomy than the P4.

#### 5.1.3 Privacy Concerns

Privacy concerns are especially salient because the data we are trying to access (patients' medical preferences) is very sensitive. Moreover, attempts to retrieve these preferences risk inadvertently exposing additional private details that the patient never intended to disclose. For example, in tracing a patient's digital footprint, we might uncover details such as sexual orientation, religious affiliation, or evidence of an extramarital affair, unintentionally disclosing this information to family or caregivers against the patient's wishes. The risks are not limited to the patient alone: much of the relevant digital footprint consists of conversations with others, which means that third parties' privacy can also be compromised. While such risks exist with P4s, they are amplified in the case of the PPR. Because the PPR presents data verbatim and links directly to original conversations, sensitive information is revealed without any ambiguity, whereas P4s may only hint at such details without confirming them outright. This dynamic underscores a difficult trade‐off: the more accurate and transparent the retrieval, the greater the potential intrusion into privacy. Mitigation strategies have been suggested, such as implementing an opt‐in mechanism for data sources [5, 17], though this faces many of the same practical challenges as advance directives. Another possibility is to require that PPR outputs be reviewed first by an impartial party bound by confidentiality, which could offer some protection while preserving the advantages of a retrieval‐based approach. Privacy concerns represent a significant ethical bottleneck for both P4s and PPRs. Although the PPR may be more exposed to these risks, this vulnerability arises as a by‐product of its greater transparency and verifiability. Any future implementation must therefore give sustained attention to developing effective strategies for mitigating these risks.

#### 5.1.4 Clarity of Decision^8^

A potential drawback of the PPR is that it leaves greater room for interpretation by clinicians and family members. This outcome is especially likely when the patient's data is mixed or inconclusive. In such cases, the PPR returns both data points supporting and against the decision, potentially placing the family and doctors in a situation of indecision. In contrast, the P4 may appear to offer a clearer, more definitive solution, which could alleviate some of the emotional burden on the family. While some may see the absence of a clear answer as a limitation of the PPR, it is actually its strength, ensuring that both supporting and opposing evidence are considered in the decision‐making process. Medical decisions, particularly in the absence of advanced directives, are by their nature challenging for families. The goal of the PPR is not to remove this burden, but to help families arrive at a decision that best reflects the patient's wishes while respecting the patient's former autonomy. By presenting the most relevant data for and against a decision in a transparent manner, the PPR aims to place the surrogate decision maker in the best possible epistemic position to make an informed choice on behalf of the patient. If the PPR returns conflicting information, this is not a fault of the system: it is fulfilling its purpose of highlighting the complexity of the patient's preferences. Hiding conflicting data behind a single, seemingly definitive response, as the P4 might do, creates a false sense of certainty about what the patient would have wanted. Ultimately, it is up to the family and clinicians, possibly with the guidance of a bioethicist, to interpret this evidence.

## 6 Conclusion

In this paper, I have shown P4s are ultimately ill‐suited to the task they are intended for. Because fine‐tuned LLM can only reproduce token‐level statistical patterns rather than capture an individual's medical values, P4s are either unnecessary and suboptimal (when relevant evidence already exists in a patient's digital footprint) or unhelpful and potentially misleading (when it does not). I have proposed an alternative, the PPR, which shifts the focus from prediction to retrieval. Rather than simulating what a patient might say, the PPR extracts and presents evidence of what the patient actually did say, together with contextual metadata. Both P4s and PPRs face technical bottlenecks, most notably in data collection and curation. If curating and processing patient data in advance is necessary, then encouraging patients to complete advance directives remains the most effective and least burdensome option [9]. Ethically, while P4s have been criticised for undermining autonomy through opacity and lack of traceability, the PPR does better on transparency and respect for autonomy by making its evidentiary basis explicit. Still, both approaches raise privacy concerns (not only for patients but also for third parties whose communications may form part of the digital footprint). In the PPR, the very features that enhance transparency also increase privacy risks. By contrast, the relative privacy protection offered by P4s is achieved only through opacity, which further undermines their legitimacy as a basis for clinical decision‐making. Further research is needed to establish how these privacy concerns can be mitigated. Although the gold standard for respecting transparency, autonomy, and privacy remains the widespread adoption of advanced directives, efforts should be focused on encouraging patients to record their preferences directly. In practice, however, some patients will inevitably face incapacitating medical decisions without such records. In these cases, AI can play a supportive role in the decision‐making process if deployed responsibly. Recent proposals have called for the development and testing of a P4 prototype [17]. I argue that such efforts should proceed with caution. Given the technical limitations of fine‐tuning and the ethical trade‐offs discussed, I argue that retrieval‐based approaches such as the PPR represent the most ethically and technically sound way to integrate AI into surrogate decision‐making. If AI systems are developed to assist decision‐making for incapacitated patients, approaches centered on transparency and data retrieval are preferable to opaque generative methods.

## Conflicts of Interest

The author declares no conflicts of interest.

## Data Availability

The author has nothing to report.

## Endnotes

1 For example the Autonomy Algorithm which I will not discuss in this paper [4].

2 For a reply on this last point see [12]. A further discussion of these can be found in 4.1.2.

3 I am grateful to Reviewer 1 for noting that the accuracy of the P4 in the absence of direct evidence in the training data remains an empirical question. Proponents might argue that if a P4 is shown to be more accurate than human surrogates, there is a prima facie case for its use. Furthermore, if such a model proves more accurate, patients or surrogates might choose to use it as a preferred decision‐making aid. However, for such a justification to be robust, the P4's performance should be measured not only against human surrogates but also against a PPR proposed in this paper. Even if the P4 were to outperform the PPR in terms of predictive accuracy, it remains an open question how to balance that accuracy against the transparency and autonomy‐preserving benefits of the PPR. Any outperformance by the P4 would likely need to be significant to justify the loss of transparency.

4 I am grateful to Reviewer 2 for suggesting I discuss this paper.

5 In Table 1 Earp et al. [5] propose five versions of the P4 based on different training data: (1) fine tuning on a personal digital footprints like emails and social media; (2) fine tuning on explicit responses to hypothetical treatment questions; (3) fine tuning on user data gathered from value‐eliciting experiments; (4) fine tuning input from surrogate decision‐makers; and (5) using a base models trained on population‐level survey/health data rather than a generic LLM. The arguments in this paper apply generally to all five versions. However, I focus primarily on the first interpretation as it is currently the most technically feasible. For the second and third proposals, the “unnecessary” dimension of the dilemma presented in this paper is particularly evident: if a user is already providing explicit ethical preferences or participating in value‐eliciting experiments, passing this data through a predictive model adds a layer of complexity that decreases transparency. In these cases, it would be more effective to access the user's responses directly or use the time the user is spending to complete these surveys to instead help the user complete formal advance directives. I am grateful to Reviewer 1 for suggesting the inclusion of these different interpretations.

6 Thanks to reviewer 2 for advising that I engage in a deeper discussion of this issue and for suggesting the inclusion of this paper.

7 Thanks to Davide Battisti for suggesting this point.

8 Thanks to Thomas Douglas and Davide Battisti for suggesting this point of discussion.

## 1

To demonstrate the feasibility of the PPR, I will provide a high‐level overview of how such a system could be implemented using embedding and vector search.^9^ This is just one possible approach (there may be other viable designs; for example, using LLM‐as‐a‐judge to assess and retrieve relevant information). I am not claiming that this is the best or only method. The purpose of this section is simply to show that a PPR is at least as technically feasible as a P4. Further empirical research would be needed to determine the most effective implementation and to evaluate its performance in real medical settings.

At the core of my proposal for the PPR is vector search, a data retrieval technique that finds items based on their semantic similarity rather than just keyword matches [22]. It works by converting data (such as text, images, or audio) into numerical representations called vectors. A vector is a list of numbers that encodes features of a piece of data in a multi‐dimensional space. For example, a sentence can be mapped to a high‐dimensional vector such that semantically similar sentences are positioned close together in the vector space. This transformation process is known as embedding [23, 24, 25]. These vectors can then be mathematically compared to find the most semantically similar items. In a typical vector search pipeline, the data is first embedded into vectors, which are stored in a vector database (VDB). Then items that are closest in meaning to a given query are retrieved by comparing their vector representations using similarity metrics. This involves calculating how ‘close’ the query vector is to other vectors in the high‐dimensional space, where proximity reflects semantic similarity. (for a survey on different VDBs and retrieval techniques see Han et al. [26]) Unlike traditional keyword search, which matches specific words, vector search can identify related ideas even if the original terms aren't used. For example, a query like “causes of the French Revolution” might return not only documents containing that exact phrase, but also texts discussing “economic inequality in 18th‐century France,” “the fall of the monarchy,” or “public discontent under Louis XVI.” This is because vector search retrieves items based on semantic proximity, enabling discovery across variations in language and expression. Vector search and the use of embeddings have already found widespread application in real‐world systems. For example, vector representations are used in large‐scale image recognition and retrieval systems to enable efficient comparison of high‐dimensional features [27]. Similarly, modern recommendation engines rely heavily on vector embeddings to capture semantic relationships across diverse data types [28, 29]. Recent work also shows promising improvements in vector search, specifically improving retrieval accuracy on large‐scale document collections and more nuanced tasks [30]. Given the rapid pace of development in vector databases and vector search technologies, we can expect these capabilities to improve further. This growing body of research indicates that implementing a PPR based on vector search is within the realm of technical possibility.

Below is a 8 step high level outline of how we can use vector embedding and search to implement a PPR:

1. Gather all relevant patient‐related data sources. Preprocess and clean the data.
2. Break the collected data into manageable chunks. Use LLM to avoid splitting conversations or documents mid‐thought. Attach metadata (timestamps, original IDs etc.) to maintain traceability.
3. Convert each data chunk into a vector using a pre‐trained embedding model.
4. Store the vectors along with their metadata in a VDB optimized for similarity search and fast retrieval.
5. Define the specific medical decision or question at hand. This should typically be input by clinicians. Use a LLM to transform the question into pro and con perspectives. For example in Alice's case, the question is “Would the patient want to be kept alive with severe brain damage?” and the two perspectives are pro: “I want to be kept alive with severe brain damage” con: “I do not want to be kept alive with severe brain damage”.
6. Embed the two pro/con query statements using the same embedding model as used for the patient data to maintain vector space alignment.
7. Perform vector similarity search to find the most relevant patient data chunks that align with each query perspective (top N similar vectors). Collect these chunks with their metadata as supporting evidence separated into pros and cons.
8. Finally use a LLM to format and present the evidence, using the metadata for each chunk to link back to the original piece of data.
